# Targeting histone deacetylase in cardiac diseases

**DOI:** 10.3389/fphys.2024.1405569

**Published:** 2024-06-24

**Authors:** Jiao Lu, Sichong Qian, Zheng Sun

**Affiliations:** ^1^ Department of Medicine, Division of Endocrinology, Diabetes, and Metabolism, Baylor College of Medicine, Houston, TX, United States; ^2^ Department of Molecular and Cellular Biology, Baylor College of Medicine, Houston, TX, United States

**Keywords:** histone deacetylase, histone deacetylase inhibitor, cardiac hypertrophy, myocardial infarction, ischemic reperfusion, heart failure, genetic mouse models, multi-omics

## Abstract

Histone deacetylases (HDAC) catalyze the removal of acetylation modifications on histones and non-histone proteins, which regulates gene expression and other cellular processes. HDAC inhibitors (HDACi), approved anti-cancer agents, emerge as a potential new therapy for heart diseases. Cardioprotective effects of HDACi are observed in many preclinical animal models of heart diseases. Genetic mouse models have been developed to understand the role of each HDAC in cardiac functions. Some of the findings are controversial. Here, we provide an overview of how HDACi and HDAC impact cardiac functions under physiological or pathological conditions. We focus on *in vivo* studies of zinc-dependent classical HDACs, emphasizing disease conditions involving cardiac hypertrophy, myocardial infarction (MI), ischemic reperfusion (I/R) injury, and heart failure. In particular, we review how non-biased omics studies can help our understanding of the mechanisms underlying the cardiac effects of HDACi and HDAC.

## Introduction

The classic zinc-dependent histone deacetylase (HDACs) are categorized into class I, class II (IIa & IIb), and class IV based on the sequence homology (Yang and Seto, 2008). HDAC1, 2, 3, 8 are class I HDACs. HDAC4, 5, 7, 9, are class IIa HDACs. HDAC6 and HDAC10 are class IIb HDACs. HDAC11 is class IV HDAC. The NAD-dependent class III HDACs are sirtuins with distinct catalytic mechanisms, which will not be discussed in this review. Based on the chemical structure, HDAC inhibitors (HDACi) of zinc-dependent HDACs can be categorized into several families (Yang and Seto, 2008). Trichostatin A (TSA), givinostat (ITF2357), CG200745, MPT0E014, and vorinostat (SAHA) belong to the hydroxamic acid chemical class and are pan-HDAC inhibitors that inhibit all classic HDACs. MS275/Entinostat, mocetinostat, and N-(2-aminophenyl)-4-{[benzyl(2-hydroxyethyl)amino]methyl} benzamide (K-183) belong to the benzamide class and inhibit class I HDACs. Valproic acid (VPA) and butyrate belong to the short-chain fatty acid class, which inhibit class I and II HDACs (Kitagawa et al., 2007; Lai et al., 2012; Maes et al., 2013; Jung et al., 2017) (Table 1). HDACi have shown the potential to attenuate pathological cardiac remodeling as a promising therapeutic strategy.

**TABLE 1:** Chemical class and HDAC targets of cited HDACi.

HDACi	Chemical class	HDAC targets
Trichostatin A (TSA)	Hydroxamic acid	Pan HDACi
Givinostat (ITF2357)	Hydroxamic acid	Pan HDACi
Vorinostat (SAHA)	Hydroxamic acid	Pan HDACi
CG200745	Hydroxamic acid	Pan HDACi
MPT0E014	Hydroxamic acid	Pan HDACi
MS275/Entinostat	Benzamide	Class I
Mocetinostat	Benzamide	Class I
K183	Benzamide	Class I
Valproic acid (VPA)	Short-chain fatty acid	Class I and II
Butyrate	Short-chain fatty acid	Class I and II

Genetic mouse models have been constructed to study the roles of each HDAC, including cardiomyocyte-specific HDAC depletion, whole-body or non-cardiac HDAC loss-of-function, HDAC transgenic overexpression, and the virus-based knockdown (KD) or overexpression (OE) of HDACs. This review focuses on how HDACi administration or HDAC genetic manipulation affects cardiac and vascular function in healthy and diseased conditions *in vivo*. We focus on disease conditions involving cardiac hypertrophy, myocardial infarction (MI), ischemic reperfusion (I/R) injury, and heart failure. In particular, we review how non-biased omics studies can help our understanding of the mechanisms underlying the cardiac effects of HDACi and HDAC.

## HDACi in cardiovascular diseases

TSA shows cardioprotective effects in several studies. TSA is protective against ischemia injury of the heart. Chronic TSA treatment reduced myocardial infarct size and improved contractile function in mice with MI (Zhang et al., 2012a; Zhang et al., 2012b) (Table 2). Acute TSA administration 24 h before cardiac ischemia alleviated HDAC4 OE-induced infarct size, cell death, and contractile dysfunctions of the mice (Zhang L. et al., 2018). TSA protects the mouse heart from the doxorubicin-induced increase in left ventricle internal diameter (LVID) and impairment in ejection fraction (EF) (Song et al., 2018).

**TABLE 2:** The effects of HDACi on cardiac functions.

HDACi	Route	Dose	Duration	Disease model	HDACi effects (vs. vehicle)	References
TSA	i.p.	0.1 mg/kg	once, 24 h before I/R	I/R in mice	alleviated HDAC4 OE-induced adverse effects in infarct size, cell death, and contractile dysfunctions	Zhang et al. (2018a)
TSA	i.p.	50 mg/kg, once per day	4 weeks, daily	Dox-induced cardiomyopathy	decrease LVID and cytoplasmic vacuolization, increase EF/FS	Song et al. (2018)
TSA	i.p.	0.6 mg/kg, once per day	9 days, daily	Vitamin D3-induced vascular calcification	increase aortic calcification	Kwon et al. (2016)
TSA	s.c.	0.6 mg/kg, twice per day	4 weeks, daily	TAC-induced cardiac hypertrophy in mice	higher FS, lower NF-κB target genes	Ooi et al. (2015)
TSA	i.p.	50 mg/kg, once per day	8 weeks, daily	Ang II-induced cardiac dysfunction in mice	increase EF	Demos-Davies et al. (2014)
TSA	i.p.	0.1 mg/kg, once per day	8 weeks, daily	LAD occlusion-induced MI in mice	smaller infarction size, better myocardial viability, improved contractile function	Zhang et al. (2012a)
TSA	i.p.	0.1 mg/kg, once daily	8 weeks, daily	LAD occlusion-induced MI in mice	better survival, smaller infarction size, better myocardial viability, improved contraction, more angiogenesis	Zhang et al. (2012b)
TSA	i.p.	0.6 mg/kg, once per day	14 days, daily	HopX OE mice with cardiac hypertrophy	lower fibrosis, less atrial arrhythmia after electrophysiologic stimulation	Liu et al. (2008)
TSA	i.p.	1 mg/kg, once per day	3 weeks, daily	TAC-induced cardiac hypertrophy in mice	higher FS, ESPVR, dP/dt; smaller heart and cardiomyocyte size	Kong et al. (2006)
TSA	i.p.	0.6 mg/kg, once per day	14 days, daily	ISO-induced cardiac hypertrophy in mice	smaller heart	Kook et al. (2003)
SAHA	i.p.	50–100 mg/kg, four times	1 day before the surgery q12h, the day of surgery, at the time of reperfusion	I/R in mice	lower ROS levels, higher mitochondrial content	Yang et al. (2019)
SAHA	drinking water	75 mg/kg per day	4 weeks, daily	TAC-induced cardiac hypertrophy	less fibrosis, less myocardial passive stiffness, no change in EF	Renaud et al. (2015)
ITF2357	diet	50 mg/kg/day	4 weeks, daily	UNX/DOCA-induced diastolic dysfunction	improved diastolic functions (E/A, E/E′, IVRT, LVEDP); less fibrosis and LV stiffness; reduced TGFβ signaling	Travers et al. (2021)
ITF2357	oral gavage or diet	3 or 30 mg/kg, once per day	10 weeks, daily	HFpEF induced by high salt in Dahl salt-sensitive rats	improved diastolic function (E/A, E’/A′, IVRT, LVEDP); no change in hypertrophy or fibrosis, attenuated the impairment in myofibril relaxation	Jeong et al. (2018)
CG200745	s.c.	1.25 or 5 mg/kg, once per day	4 weeks, daily	UNX/DOCA-induced cardiac hypertrophy	improved EF, LVID, LVPW, IVS	Lee et al. (2019)
CG200745	s.c.	1.25 or 5 mg/kg, once per day	4 weeks, daily	DOCA-induced hypertrophy in rats	lower SBP, heart weight, fibrosis size, and fibrotic gene expression	Lee et al. (2016)
MPT0E014	gavage	50 mg/kg, once per day	7 days, daily	ISO-induced HF in rats	improved FS, lower IL6, and TGFβ	Lkhagva et al. (2015)
MPT0E014	gavage	100 mg/kg, once per day	7 days, daily	ISO-induced HF in mice	lower LVEDD, higher EF/FS, lower fibrosis, ANP, and TGFβ	Kao et al. (2013)
K-183	i.p.	40 mg/kg, twice per day	3 days, daily	ISO-induced cardiac hypertrophy in rats	higher cardiac output, but higher collagen deposition in LV, no change in hypertrophy (6 mg/kg TSA no benefits)	Kitagawa et al. (2007)
Mocetinostat	i.p.	10 mg/kg, once per day	4 weeks, every other day	TAC-induced cardiac hypertrophy in rats	lower systolic blood pressure, LVPW, IVS, and heart weight; less fibrosis	Kim et al. (2021)
Mocetinostat	c-Kit + cell transplant	2 µM for cultured cells	cell infusion after MI	LAD occlusion-induced MI in rats	lower LVEDP, less collagen deposition, lower IL6	Zakharova et al. (2015)
Mocetinostat	i.p.	20 mg/kg, once per day	3 weeks, daily	LAD occlusion-induced MI in rats	improved EF, cardiac output, and dP/dt, reduced LVEDP; less fibrosis, lower IL6	Nural-Guvener et al. (2015)
MS275/Entinostat	s.c.	0.1 g per day	10 days, daily	rapid ventricular pacing in rabbits	alleviated prolongation of repolarization, no change in FS, less fibrosis	Freundt et al. (2019)
MS275/Entinostat	i.p.	10 mg/kg	twice, 24 h and 1 h before I/R	I/R in rats	improved dP/dt, smaller infarct area (TSA and TubA show no benefits)	Aune et al. (2014)
Butyrate	drinking water	220 mg/kg per day	2 weeks, daily	hypoxia-induced pulmonary hypertension in rats	attenuated RV hypertrophy, RV systolic pressure, pulmonary vascular remodeling	Karoor et al. (2021)
Butyrate	drinking water	1%	12 weeks, daily	HFD-induced cardiac dysfunction in mice	lower body weight, blood glucose, insulin, and TG levels; improved EF, LVID, and LVPW; less fibrosis	Zhang et al. (2017)
Butyrate	drinking water	1%	12 weeks, daily	STZ-induced diabetes in mice	improved EF, reduced fibrosis and apoptosis, enhanced angiogenesis	Chen et al. (2015)
VPA	drinking water	0.71%	4 weeks, daily	TAC-induced cardiac hypertrophy in rats	lower SBP and heart weight; improved IVS, LVID, LVPW, EF, less fibrosis; improved relaxation in the ascending aorta	Jung et al. (2019)
VPA	i.p.	250 mg/kg, twice per day	60 min-pre and 12 h-post of I/R	I/R in rats	improved EF and FS, smaller infarct size, suppressed inflammation genes	Tian et al. (2019)

Intraperitoneal injection (i.p.), ischemia/reperfusion (I/R), overexpression (OE), doxorubicin (Dox), left ventricular internal dimension (LVID), ejection fraction (EF), fractional shortening (FS), subcutaneous injection (s.c.), transverse aortic constriction (TAC), angiotensin II (Ang II), left anterior descending artery (LAD), end-systolic pressure-volume relation (ESPVR), pressure change over time (dP/dt), isoproterenol (ISO), reactive oxygen species (ROS), right ventricular (RV), high fat diet (HFD), triglyceride (TG), left ventricular posterior wall (LVPW), streptozotocin (STZ), uninephrectomy (UNX), deoxycorticosterone acetate (DOCA), the ratio of peak early to late diastolic filling velocity (E/A), the ratio of early mitral inflow velocity and mitral annular early diastolic velocity (E/E′), isovolumetric relaxation time (IVRT), left ventricular end-diastolic pressure (LVEDP), left ventricular (LV), transforming growth factor β (TGFβ), heart failure with preserved ejection fraction (HFpEF), the ratio of peak diastolic tissue velocity (E′/A′), interventricular septum (IVS), systolic blood pressure (SBP), heart failure (HF), left ventricular end diastolic diameter (LVEDD), atrial natriuretic peptide (ANP), myocardial infarction (MI), left ventricular end-diastolic volume (LVEDV), left ventricular end-systolic volume (LVESV).

The cardioprotective effects of TSA have also been investigated in cardiac hypertrophy models. Angiotensin II (Ang II)-induced EF decline in mice was abolished by treatment of TSA (Demos-Davies et al., 2014) (Table 2). TSA also reduced atrial arrhythmia duration after electrophysiologic stimulation and reduced atrial fibrosis in mice overexpressing homeodomain-only protein, a transgenic model of cardiac hypertrophy (Liu et al., 2008). In transverse aortic constriction (TAC)-induced cardiac hypertrophy in mice, TSA preserved fractional shortening (FS), the end-systolic pressure-volume relation, and the pressure change over time (dP/dt), as well as inhibited cardiac hypertrophy (Kong et al., 2006). TSA attenuated the increase in wall thickness and prevented FS decline induced by TAC, as well as suppressed NFκB target genes (Ooi et al., 2015). This is in line with the attenuated cardiac hypertrophy induced by isoproterenol infusion (Kook et al., 2003). While most studies have shown that TSA is protective for the heart, its effect appears opposite in the vascular system. A study reported that TSA significantly enhanced Pi-induced calcium deposition in the aorta and enhanced vitamin D3-induced vascular calcification (Kwon et al., 2016).

SAHA was studied in I/R or hypertrophic mouse models. In I/R injury, SAHA decreased the loss of mitochondrial membrane potential and decreased cytosolic reactive oxygen species (ROS) levels (Yang et al., 2019) (Table 2). In another study, SAHA reduced fibrosis and passive stiffness of the left ventricle myocardium after TAC, but had no effect on cardiac hypertrophy and systolic function (Renaud et al., 2015). ITF2357 (givinostat) can improve diastolic function in models of diastolic dysfunction, including the uninephrectomy (UNX)/deoxycorticosterone acetate (DOCA)-induced diastolic dysfunction model, and the heart failure with preserved ejection fraction (HFpEF) model induced by high salt in Dahl salt-sensitive rats (Jeong et al., 2018; Travers et al., 2021).

Cardiac effects have been characterized for several new HDACis, including CG200745, MPT0E014, and K-183. The pan-HDACi CG200745 attenuated cardiac hypertrophy and fibrosis in DOCA-induced hypertensive rats (Lee et al., 2016; Lee et al., 2019). MPT0E014 improved systolic function and lowered TGFβ levels in isoproterenol-induced heart failure in both mice and rats (Kao et al., 2013; Lkhagva et al., 2015). In isoproterenol-induced cardiac hypertrophy in rats, K-183 attenuated the decrease of cardiac output, even though it did not ameliorate cardiac hypertrophy or collagen production. In comparison, TSA did not show beneficial effects in this study (Kitagawa et al., 2007).

Mocetinostat, a class I HDACi, attenuated cardiac hypertrophy in a TAC-induced hypertrophic model in rats (Kim et al., 2021) (Table 2). It also improved cardiac function in rats with MI, including improved EF and decreased scar size and fibrosis (Nural-Guvener et al., 2015). Furthermore, infusion of Mocetinostat treated c-Kit + cells resulted in a better outcome in myocardial infarcted rats, with reduced left ventricular end-diastolic pressure (LVEDP) and less collagen deposition (Zakharova et al., 2015). Another class I HDACi MS275/Entinostat alleviated the prolonged repolarization in rabbits subjected to rapid ventricular pacing (Freundt et al., 2019). In addition, Entinostat improved dP/dt and reduced infarct size in I/R hearts from rats, whereas TSA and TubA did not show protection (Aune et al., 2014).

Butyrate and VPA, as common class I & II HDACi, were investigated in various disease conditions. In hypoxia-induced pulmonary hypertension in rats, treatment with butyrate attenuated right ventricular (RV) hypertrophy, systolic pressure increase in RV, and pulmonary vascular remodeling (Karoor et al., 2021). In high-fat diet (HFD)-induced cardiac dysfunction in mice, treatment with butyrate lowered body weight, blood glucose, insulin, and TG levels, attenuated cardiac hypertrophy, and improved contractile function (Zhang et al., 2017). Butyrate improved EF, reduced fibrosis and apoptosis, and enhanced angiogenesis in streptozotocin (STZ)-induced diabetic mice (Chen et al., 2015). VPA considerably attenuated systolic blood pressure and hypertrophy, improved cardiac function, and improved relaxation in the ascending aorta in TAC-induced cardiac hypertrophy in rats (Jung et al., 2019) (Table 2). VPA also improved cardiac function and reduced infarct size in rats with I/R injury (Tian et al., 2019). In summary, most studies on HDACis reported protective effects of HDACis on cardiac function. The cardioprotective effects of HDACis are widely observed in cardiac hypertrophy, MI, I/R, and heart failure.

### Cardiomyocyte-specific HDAC genetic deletion

HDACi can have many systemic effects or HDAC-independent effects. Genetic mouse models with cardiomyocyte-specific HDAC loss-of-function have served as a valuable tool for studying the functions of HDACs in the heart. Cardiac-specific double knockout (KO) of HDAC1 and HDAC2 with α-myosin heavy chain (αMHC)-Cre in mice caused postnatal lethality, cardiac arrhythmias, severe ventricular dilation, and heart failure (Montgomery et al., 2007) (Table 3). In comparison, a single gene deletion of either HDAC1 or HDAC2 in the heart with αMHC-Cre displayed no gross cardiac abnormalities (Montgomery et al., 2007). HDAC1 KD by intramyocardial small interfering RNA (siRNA) injection showed recovered cardiac function, improved myocardial injury and inflammation, and less cardiomyocyte apoptosis in a septic mouse model (Nong et al., 2022) (Table 3). Similarly, KD of HDAC1 via intravenous injection of adeno-associated virus (AAV)-siRNA carrying cardiomyocyte-specific cTNT (cardiac troponin T) promotor improved cardiac function, restored lipid contents, and reduced myocardial injury as well as inflammation in a coronary heart disease model of rats (Zhou et al., 2022). Cardiomyocyte-specific KO of HDAC2 with αMHC-Cre alleviated ultrastructural disorganization of atrial myocytes in a disease model of atrial fibrillation (Scholz et al., 2019). Cardiomyocyte-specific KO of HDAC3 with αMHC-Cre in mice caused cardiac hypertrophy, elevated myocardial triglyceride content, and lethal heart failure (Montgomery et al., 2008) (Table 3). In contrast, postnatal myocyte-specific KO of HDAC3 with myosin creatine kinase (MCK)-Cre only displayed lethal heart failure on HFD (Sun et al., 2011). Both studies suggest that HDAC3 is critical in regulating cardiac hypertrophy and lipid metabolism. The role of HDAC3 during heart development has also been investigated. Mouse embryos depleted for HDAC3 in the second heart field with Isl1-Cre [mainly in RV and outflow tract (Cai et al., 2003)] or Mef2C-Cre [in anterior heart field (Verzi et al., 2005)] is lethal, along with ascending aortic dilatation, double outlet right ventricle, and aberrant valve development (Lewandowski et al., 2015). HDAC3 KO in the developing epicardium with Wt1-Cre led to hypoplasia of the ventricular myocardial wall and reduced epicardium-derived cells in mice, suggesting that HDAC3 promotes myocardial growth during development (Jang et al., 2022).

**TABLE 3:** The effects of HDAC genetic manipulations on cardiac functions.

Genetic model	Cardiac phenotype (KO/KD/OE/KI vs. WT in the same stress/disease model)	References
cardiomyocyte (CM) or myocyte-specific loss-of-function: knockout (KO) or knockdown (KD)		
CM-specific KO of HDAC1/2 (αMHC-Cre)	postnatal lethality, arrhythmias, severe ventricular dilatation, heart failure; no phenotype from single deletion	Montgomery et al. (2007)
HDAC1 KD by intramyocardial siRNA injection	improved cardiac function and pathology, less cardiomyocyte apoptosis in the sepsis model	Nong et al. (2022)
KD of HDAC1 via iv AAV-siRNA in rats (cTNT)	improved ventricular function; less myocardial TG, injury, and inflammation in a coronary heart disease model	Zhou et al. (2022)
CM-specific KO HDAC2 (αMHC-Cre)	alleviated ultrastructural disorganization of myocytes in a disease model of atrial fibrillation	Scholz et al. (2019)
CM-specific KO of HDAC3 (αMHC-Cre)	cardiac hypertrophy, elevated myocardial TG content, lethal heart failure	Montgomery et al. (2008)
Postnatal myocyte-specific KO of HDAC3 (MCK-Cre)	cardiac hypertrophy and lethal heart failure only on a high-fat diet	Sun et al. (2011)
HDAC3 KO in the 2nd heart field (Isl1- or Mef2C-Cre)	embryonic lethality, ascending aortic dilatation, double outlet right ventricle, aberrant valve development	Lewandowski et al. (2015)
HDAC3 KO in developing epicardium (Wt1-CreERT2)	ventricular myocardial wall hypoplasia, reduced epicardium-derived cells	Jang et al. (2022)
myocyte-specific KO of HDAC4 (αMHC-Cre)	higher basal ANP gene expression but fails to be induced in response to elevated preload	Hohl et al. (2013)
CM-specific KO of HDAC4 (αMHC-Cre)	reduced exercise capacity, transiently impaired cardiac function after exercise, no phenotype after TAC	Lehmann et al. (2018)
CM-specific KO of HDAC4 (αMHC-Cre)	heart failure in diabetes models (STZ or db/db), rescued by HDAC4 NT (N-terminus) expression	Kronlage et al. (2019)
transplant of siHDAC4-treated CSCs in MI hearts	improved ventricular function, enhanced CSC-derived regeneration and neovascularization; opposite to OE	Zhang et al. (2014)
HDAC9 KD by intracoronary adenovirus of shRNA	improved ventricular contraction and dilation, better cell viability, smaller infarct size in MI	Liu et al. (2022)
whole-body or non-cardiac loss-of-function		
VSMC-speicifc KO of HDAC1 (SM22α-Cre)	exacerbate vascular calcification	Kwon et al. (2016)
whole-body KO of HDAC1	embryonic lethal	Montgomery et al. (2007)
whole-body KO of HDAC2	perinatal lethality, cardiac defects, loss of ventricular lumen, no steatosis or fibrosis in neonate myocardium	Montgomery et al. (2007)
whole-body KO of HDAC2	partial postnatal lethality and perinatal defects, attenuated hypertrophy on ISO, Hod OE, or TAC in adults	Trivedi et al. (2007)
HDAC3 KD via i.v. injection of AAV for shRNA	reduced infarct size in diabetic rats after myocardial ischemia-reperfusion injury	Qiu et al. (2021)
HDAC3 KD via i.v. injection of AAV for shRNA	thicker ventricular wall, smaller ventricular diameter, better EF, smaller CM size, less fibrosis/apoptosis in MI	Na et al. (2021)
myeloid-specific KO of HDAC3 (LysM-Cre)	stabilized and more collagen deposition in atherosclerotic plaques, more anti-inflammatory macrophages	Hoeksema et al. (2014)
neural crest-specific KO of HDAC3 (Wnt1/Pax3-Cre)	perinatal lethality, aortic and ventricular defect	Singh et al. (2011)
whole-body KO of HDAC4	skeletal abnormality; no apparent abnormalities of the heart by histological examination	Vega et al. (2004)
HDAC4 KD via i.v. injection of lentiviral shRNA	alleviated vascular inflammation after Ang II treatment	Yang et al. (2018)
whole-body KO of HDAC5	exacerbated hypertrophy on TAC or calcineurin, similar phenotype to WT on chronic beta-adrenergic stimulation	Chang et al. (2004)
whole-body KO of HDAC5/9	lethal ventricular septal defects, myocardial wall thinning	Chang et al. (2004)
whole-body KO of HDAC9	exacerbated hypertrophy after TAC or constitutive cardiac activation of calcineurin	Zhang et al. (2002)
whole-body KO of HDAC9	alleviated aortic calcification, improved survival in a disease model of vascular calcification	Malhotra et al. (2019)
VSMC-specific KO of HDAC9 (Tagln-cre)	alleviated neointimal hyperplasia and stenosis in an arterial stenosis model	Cardenas et al. (2019)
endothelial-specific KO of HDAC9 (Cdh5-Cre)	reduced plaque area and plaque lipid content, increased fibrous cap thickness in an atherosclerosis model	Lecce et al. (2021)
whole-body KO of HDAC6	alleviated cardiac contractile dysfunction after Ang II or TAC, similar hypertrophy, or even worse fibrosis	Demos-Davies et al. (2014)
whole-body KO of HDAC6	increased myofibril stiffness, exacerbated diastolic dysfunction in response to hypertension or aging	Lin et al. (2022)
whole-body KO of HDAC6	alleviated doxorubicin cardiotoxicity, conserved cardiac function after doxorubicin	Song et al. (2018)
HDAC6 KD via ventricular shRNA in rats	alleviated cardiac dysfunction and dilation, suppressed inflammasome and CM apoptosis in a DCM model	Pang et al. (2022)
whole-body KO of HDAC11	lower adiposity, CM apoptosis, cardiac inflammation, and oxidative stress on fructose diet; higher BP	Fan et al. (2018)
gain-of-function: overexpression (OE) or knock-in (KI) of an active form		
HDAC1/2 OE in CM via AAV (TNNT2)	improved cardiac function in an Eed^CKO^ model of DCM	Ai et al. (2017)
CM-specific transgenic OE of HDAC2 (αMHC)	cardiac hypertrophy	Trivedi et al. (2007)
CM-specific transgenic OE of HDAC2 (αMHC)	larger heart, higher ANP	Eom et al. (2011)
whole-body HDAC2 C262A/C274A knock-in	resistant to diastolic dysfunction on saltwater/nephrectomy/aldosterone or mild TAC	Yoon et al. (2021)
CM-specific transgenic OE of HDAC3 (αMHC)	ventricular myocardium thickening at birth due to CM hyperplasia, similar hypertrophy to WT in adults on ISO	Trivedi et al. (2008)
Expression of HDAC8 via i.v. injection of AAV	alleviated cardiac hypertrophy and contractile dysfunction after miR-21-3p OE	Yan et al. (2015)
CM-specific transgenic OE of HDAC4 (αMHC)	cardiac dysfunction, larger heart and myocyte size, interstitial fibrosis, exacerbated cardiac dysfunction in MI	Zhang et al. (2018b)
CM-specific transgenic OE of HDAC4 (αMHC)	larger infarct size, impaired ventricular functional recovery following I/R injury	Zhang et al. (2018a)
Cardiac OE of HDAC4-NT (N-terminus) by AAV (Myl2)	alleviated heart failure on TAC, no effects on intra-cardiomyocyte lipid accumulation	Lehmann et al. (2018)
CM-specific transgenic OE of active HDAC6(NT-allD) (αMHC)	S/T-to-D mutant of the terminal (HDAC6 NT-all D), exacerbated atrial fibrillation and interstitial fibrosis	Sawa et al. (2021)

Adeno-associated virus (AAV), triglyceride (TG), atrial natriuretic peptide (ANP), transverse aortic constriction (TAC), streptozotocin (STZ), cardiac stem cells (CSC), short hairpin RNA (shRNA), vascular smooth muscle cell (VSMC), isoproterenol (ISO), angiotensin II (Ang II), wildtype (WT), dilated cardiomyopathy (DCM), blood pressure (BP), embryonic ectoderm development inactivation in the postnatal heart (EedCKO).

Mice with cardiac-specific HDAC4 depletion using αMHC-Cre show elevated basal cardiac atrial natriuretic peptide (ANP) gene expression but a failure of the ANP gene expression activation by cardiac pressure overload (Hohl et al., 2013) (Table 3). The cardiac HDAC4 KO mice also show reduced exercise capacity and transiently impaired cardiac function after exercise but have normal phenotypic changes in response to pressure overload (Lehmann et al., 2018). In another study, mice with cardiac KO of HDAC4 developed heart failure in diabetes models (STZ or db/db), which was rescued by the expression of the N-terminal proteolytic fragment of HDAC4 (Kronlage et al., 2019), suggesting that the N-terminal fragment of HDAC4 plays a role in protecting the diabetic heart from failure. In contrast, transplanting siHDAC4-treated cardiac stem cells (CSC) in MI hearts improved cardiac function and enhanced CSC-derived myocardial regeneration and neovascularization, which is opposite to the effects caused by HDAC4 overexpression (Zhang et al., 2014). HDAC9 KD by intracoronary delivery of adenoviruses carrying HDAC9-short hairpin RNA (shRNA) restored cardiac function in MI and reduced infarct size (Liu et al., 2022). In summary, most studies using HDAC cardiac-specific KO mouse models reported detrimental effects on the heart, whereas virus-mediated KD can improve cardiac function only under specific conditions.

### Whole-body or non-cardiac HDAC depletion

While whole-body KO of HDAC1 is embryonic lethal, whole-body KO of HDAC2 (exons 2-4 deletion) is perinatal lethal, with abnormal morphology of right ventricle, interventricular septum thickening, and bradycardia (Montgomery et al., 2007) (Table 3). In another mouse model of HDAC2 whole-body KO (exons 9–14 deletion), partial lethality was observed along with thickened ventricular wall perinatally. However, when HDAC2 KO mice were subject to hypertrophy stimuli, there was attenuated hypertrophy compared to wild type (WT) in response to isoproterenol, homeobox gene Hod overexpression, or pressure overload. In contrast, transgenic overexpressing HDAC2 in cardiomyocytes led to cardiac hypertrophy, indicating that HDAC2 is both necessary and sufficient for hypertrophic responses (Trivedi et al., 2007). In the aforementioned HDAC2 whole-body KO models, both exons 2-4 deletion of HDAC2 and exons 9–14 deletion target the catalytic domain that is required for the enzymatic activity of HDAC2. The lethality and hypertrophy observed in both models suggest that the deacetylase activity of HDAC2 is critical for the normal function of the heart. In addition to the whole-body KO of HDAC1, the vascular-specific role of HDAC1 was also investigated. Vascular smooth muscle cell (VSMC)-specific KO of HDAC1 by SM22α-Cre exacerbated vascular calcification (Kwon et al., 2016). This study suggests that targeting HDAC1 in vasculature may be a therapeutic strategy for calcification-induced vascular diseases. HDAC3 KD via intravenous injection of AAV-shRNA in STZ-induced diabetic rats reduced infarct size after myocardial I/R injury (Qiu et al., 2021). Similarly, another study found that HDAC3 KD, through intravenous injection of AAV-shRNA, attenuated heart failure induced by MI in mice (Na et al., 2021). In addition to the systemic KD of HDAC3, the tissue-specific KO of HDAC3 also provides insights into the function of HDAC3 in the heart and vascular system. Myeloid-specific KO of HDAC3 with LysM-Cre resulted in stabilized atherosclerotic plaques and more anti-inflammatory macrophages (Hoeksema et al., 2014). Neural crest-specific KO of HDAC3 with Wnt1/Pax3-Cre caused perinatal lethality associated with aortic and ventricular defects (Singh et al., 2011).

Whole-body KO of HDAC4 resulted in skeletal abnormality but no obvious morphological abnormalities of the heart (Vega et al., 2004) (Table 3). HDAC4 KD in mice via intravenous injection of lentiviral shRNA alleviated vascular inflammation after Ang II treatment, indicating that HDAC4 may be a therapeutic target for vascular inflammatory diseases (Yang et al., 2018). In contrast to the not obvious cardiac phenotype by whole-body HDAC4 KO, the changes by HDAC5 or HDAC9 whole-body KO were more evident. Whole-body KO of HDAC5 alone exacerbated hypertrophy upon TAC or calcineurin activation in mice (Chang et al., 2004). Similarly, mice with whole-body KO of HDAC9 alone developed cardiac hypertrophy by 8 months old and are more susceptible to pressure overload-induced hypertrophy (Zhang et al., 2002). These two studies suggest the important role of HDAC5 and HDAC9 in suppressing hypertrophic signaling in the heart. In addition, whole-body KO of both HDAC5 and HDAC9 worsened the outcome, resulting in lethal ventricular septal defects and myocardial wall thinning (Chang et al., 2004). In summary, these studies suggest the redundant functions of HDAC5 and HDAC9 in controlling cardiac development and function. In terms of the role of HDAC9 in the vasculature, one study reported that whole-body HDAC9 KO alleviated aortic calcification and improved survival in a disease model of vascular calcification (Malhotra et al., 2019). VSMC-specific HDAC9 KO with Tagln-Cre alleviated neointimal hyperplasia and stenosis in an arterial stenosis model by carotid artery ligation (Cardenas et al., 2019). Endothelial-specific KO of HDAC9 with Cdh5-Cre showed reduced plaque area and plaque lipid content, and increased fibrous cap thickness in an atherosclerosis model, suggesting that targeting HDAC9 may aid in the treatment of atherosclerosis (Lecce et al., 2021) (Table 3). These three studies provide evidence of a beneficial outcome by depleting HDAC9 in the vasculature, which is opposite to that in the heart.

Whole-body KO of HDAC6 preserved cardiac systolic function in response to Ang II or TAC, despite cardiac hypertrophy (Demos-Davies et al., 2014) (Table 3). HDAC6 KD in rats using lentiviral shRNA injected into the left ventricular cavity also demonstrated alleviated systolic dysfunction in a dilated cardiomyopathy (DCM) model (Pang et al., 2022). However, whole-body HDAC6 KO exacerbated diastolic dysfunction in response to hypertension or aging, along with increased cardiac myofibril stiffness (Lin et al., 2022). These studies suggest that it is possible to have opposite effects on diastolic *versus* systolic functions from the same genetic manipulation. Additionally, whole-body KO of HDAC6 also alleviated doxorubicin-induced cardiotoxicity, shown by conserved cardiac function after doxorubicin treatment (Song et al., 2018). Finally, whole-body HDAC11 deficiency led to lower adiposity, cardiac inflammation and oxidative stress, but higher blood pressure on the fructose diet (Fan et al., 2018).

In summary, the widely observed detrimental effects on the heart resulting from whole-body HDAC KO align with the observations from HDAC cardiac-specific KO. Specifically, the detrimental effects on the heart are observed in whole-body KO of HDAC1, HDAC2, HDAC4, HDAC5, HDAC9, or HDAC6, as well as in cardiac-specific KO of HDAC1, HDAC2, HDAC3, or HDAC4 (Table 3). Some beneficial effects of whole-body HDAC KD are also observed in some cases. For instance, whole-body KD of HDAC3, HDAC4, or HDAC6 was shown to be protective against ischemic injury, vascular inflammation, or DCM, respectively (Yang et al., 2018; Na et al., 2021; Qiu et al., 2021; Pang et al., 2022). Cardiac-specific KD of HDAC1 or HDAC9 was found cardioprotective in a sepsis model and a coronary heart disease model, or MI model, respectively (Liu et al., 2022; Nong et al., 2022; Zhou et al., 2022). In contrast to cardiac phenotypes, a few lines of evidence suggest a protective role of HDAC depletion in the vascular system. For example, VSMC-specific KO of HDAC9 is protective in an arterial stenosis model, and endothelial cells-specific KO of HDAC9 is protective in an atherosclerosis model (Cardenas et al., 2019; Lecce et al., 2021). These results suggest the distinct roles of HDAC in the heart *versus* the vasculature.

### HDAC gain-of-function

Overexpression of HDAC1 and 2 in cardiomyocytes via intraperitoneal injection of AAV9 with TNNT2 promoter improved cardiac function in a DCM model created by depletion of EED (embryonic ectoderm development) (Ai et al., 2017) (Table 3). Cardiomyocyte-specific transgenic OE of HDAC2 with αMHC promoter resulted in cardiac hypertrophy (Trivedi et al., 2007; Eom et al., 2011). HDAC2 can be post-translationally modified by S-nitrosylation on C262A and C274A sites by neuronal nitric oxide synthase pathway, which promotes HDAC2 activity in the nucleus. Abolishing the S-nitrosylation in mice with HDAC2 C262A/C274A knock-in resulted in resistance to diastolic dysfunction induced by salt water/nephrectomy/aldosterone or mild TAC (Yoon et al., 2021). Together, these studies suggest that overexpressing HDAC2 activates hypertrophic signaling. Cardiomyocyte-specific transgenic OE of HDAC3 with αMHC displayed ventricular myocardium thickening at birth due to cardiomyocyte hyperplasia, but there was no further increase in cardiac hypertrophy in adults under isoproterenol stimulation compared with WT (Trivedi et al., 2008). This result suggests that HDAC3 regulates cardiomyocyte proliferation during heart development, and it plays distinct roles in development *versus* adulthood. While miR-21-3p suppressed cardiac hypertrophy, overexpression of HDAC8 via AAV9 attenuated its suppressing effect on hypertrophy (Yan et al., 2015). Transgenic OE of HDAC4 in cardiomyocytes with αMHC promoter impaired cardiac contraction and exacerbated contractile dysfunction on MI, as well as impaired ventricular functional recovery following I/R injury (Zhang L. et al., 2018; Zhang LX. et al., 2018), suggesting that activation of HDAC4 is a crucial regulator in ischemic injury. Cardiac OE of HDAC4-NT (N-terminus) by intravenous injection of AAV9 with the cytomegalovirus (CMV)-enhanced myosin light chain promoter (Myl2) protected against the heart failure on TAC (Lehmann et al., 2018), suggesting that HDAC4-NT can promote cardiac function. However, cardiomyocyte-specific transgenic OE of active HDAC6 with αMHC promoter exacerbated atrial fibrillation and interstitial fibrosis (Sawa et al., 2021), suggesting a role of HDAC6 catalytic activity in atrial fibrillation. In summary, HDAC OE in the heart can lead to different results depending on the specific HDAC. HDAC2, 3, and 4 are involved in hypertrophic signaling in the heart, as shown by the common phenotype of cardiac hypertrophy in response to cardiomyocyte-specific OE of HDAC2, 3 or 4 (Trivedi et al., 2007; Trivedi et al., 2008; Eom et al., 2011; Zhang L. et al., 2018).

### Omics analysis of HDACi or HDACs

Multi-omics approaches provide non-biased insights into the effects of HDACi and HDACs functions. Microarray analysis on ventricular myocardium in dogs identified transcriptomic changes in response to NVS050, mocetinostat, and SAHA. The NVS050 and mocetinostat-induced differentially expressed genes (DEGs) are enriched in protein transport/trafficking and localization, which might contribute to the delayed cardiac repolarization in response to these HDACi (Spence et al., 2016) (Table 4). Microarray on H9c2 rat cardiomyocytes treated by CBHA or TSA found canonical TGFβ, TNF-α, IFNγ, and IL-6 networks were downregulated by both HDACi (Majumdar et al., 2012). ChIP-sequencing identified increased histone acetylation of NFκB target genes in TAC-induced hypertrophic hearts, which were attenuated by TSA (Ooi et al., 2015). Mass spectrometry analysis on a mouse model of diastolic dysfunction suggested that ITF2357/Givinostat treatment suppressed cardiac fibrosis by blocking extracellular matrix deposition. RNA-sequencing further revealed suppression of cardiac fibroblast activation by ITF2357/Givinostat treatment (Travers et al., 2021). Mass spectrometry unraveled cardiac 20S acetylome in murine hearts, and HDAC inhibition via SAHA and valproate induced the acetylation of five identified lysine residues, correlating with the enhanced 20S proteasomal activity, which suggests a new strategy of improving the proteolytic function in treating heart failure (Wang et al., 2013). A novel HDACi compound 106 in a mouse model of Friedreich ataxia, a neurodegenerative and cardiac disease, reversed most DEGs in these mice to WT levels (Rai et al., 2008). Proteomics and microarray on Rhein-treated primary human ventricular cardiac fibroblasts found that Rhein inhibited collagen contraction and increased the abundance of SMAD7, indicating Rhein may serve as a potential anti-fibrotic agent to treat cardiac fibrosis (Barbosa et al., 2020). In summary, the omics studies with HDACis indicate the anti-inflammatory and anti-fibrotic role of HDACi in cardiac diseases.

**TABLE 4:** Omics analysis on HDACi or HDAC genetic manipulations in the heart.

Omics analysis	HDACi or HDAC	Cell or tissue	References
HDACi			
microarray	NVS050, vorinostat	ventricular myocardium in dogs	Spence et al. (2016)
microarray	CBHA, TSA	H9c2 rat cardiomyocytes	Majumdar et al. (2012)
ChIP-seq	TSA	LV in mice with TAC	Ooi et al. (2015)
MS proteomics	ITF2357/Givinostat	ECM fraction of LV in mice with UNX or/and DOCA	Travers et al. (2021)
RNA-seq	ITF2357/Givinostat	LV in mice with UNX or/and DOCA	Travers et al. (2021)
MS acetylome	SAHA	20S proteasome from mouse myocardium	Wang et al. (2013)
microarray	compound 106	heart in a frataxin mouse model	Rai et al. (2008)
microarray	mocetinostat	ventricular myocardium in dogs	Spence et al. (2016)
microarray	Rhein	primary human ventricular cardiac fibroblasts	Barbosa et al. (2020)
secretome proteomics	Rhein	primary human ventricular cardiac fibroblasts	Barbosa et al. (2020)
MS acetylome	Valproate	20S proteasome from mouse myocardium	Wang et al. (2013)
HDAC genetic manipulation			
microarray	cardiac deletion of HDAC1/2	mouse ventricle at P8	Montgomery et al. (2007)
microarray	KD of HDAC1 via iv AAV-shRNA injection	rat heart	Zhou et al. (2022)
microarray	cardiac HDAC3 deletion with aMHC-Cre	mouse left ventricles at 5 weeks old	Montgomery et al. (2008)
microarray	myocyte HDAC3 deletion postnatally with MCK-Cre	mouse heart at 6 weeks old	Sun et al. (2011)
microarray	HDAC3 deletion in heart field progenitor with Isl1-Cre	mouse embryonic heart at E9.5	Lewandowski et al. (2015)
RNA-Seq	HDAC3 KO in developing epicardium (Wt1-CreERT2)	mouse epicardial cells	Jang et al. (2022)
microarray	CM-specific HDAC4 KO with αMHC-Cre	mouse heart in adults after exercise	Lehmann et al. (2018)

m-carboxycinnamic acid bis-hydroxamide (CBHA), left ventricular (LV), uninephrectomy (UNX), deoxycorticosterone acetate (DOCA), transverse aortic constriction (TAC), extracellular matrix (ECM), knockdown (KD), adeno-associated virus (AAV), knockout (KO).

Microarray analysis on HDAC1 and HDAC2 depleted hearts identified that upregulated genes in KO compared with WT are those involved in cell structure and motility, particularly those encoding cytoskeletal proteins or ion channels (Montgomery et al., 2007) (Table 4). Another miRNA microarray analysis revealed that HDAC1 inhibition via shRNA led to miR-182 overexpression, which suppresses TGF-β/Smad pathway to alleviate coronary heart disease (Zhou et al., 2022). Microarray on HDAC3-depleted hearts with αMHC-Cre at 5 weeks old found DEGs in cardiac metabolism, including downregulated genes in glucose metabolism and upregulated genes in fatty acid uptake, fatty acid oxidation, and mitochondrial uncoupling (Montgomery et al., 2008). However, postnatal HDAC3 depletion in the heart with MCK-Cre showed downregulated expression of mitochondrial genes and genes in lipid metabolism (Sun et al., 2011). These two studies suggest HDAC3 is a crucial regulator of cardiac energy metabolism. During cardiac development, HDAC3 deletion in the second heart field progenitor cells activates TGF-β signaling pathway, which mediates enhanced endothelial-to-mesenchymal transition and extracellular matrix remodeling, as identified by microarray (Lewandowski et al., 2015). This result suggests that HDAC3 suppresses TGF-β in the developing heart. Additionally, RNA-seq analysis in HDAC3-deficient epicardial cells found decreased expression of FGF9 (fibroblast growth factor 9) and IGF2 (insulin-like growth factor 2), while supplementation of FGF9 or IGF2 rescued cardiomyocyte proliferation defects, suggesting that HDAC3 promotes myocardial growth by activating FGF9 and IGF2 (Jang et al., 2022). Increased Nr4a1 that activates hexosamine biosynthetic pathway was identified by microarray in HDAC4-depleted hearts after exercise, whereas the impaired exercise and cardiac function can be rescued by re-expression of HDAC4 N-terminal proteolytic fragment, suggesting a cardioprotective role of HDAC4 that may involve the regulation of the hexosamine biosynthetic pathway (Lehmann et al., 2018). In summary, the omics analysis in HDAC KO or KD hearts identified pathways involved in cardiac development or metabolism. Future work to further investigate the underlying molecular mechanisms by which HDACs regulate cardiac function will be necessary to facilitate the development of improved therapies to treat cardiac diseases.

## Conclusion

Accumulating evidence suggests cardioprotective effects of different HDACi. However, a few studies reported no change or adverse effects. For example, TSA showed no benefits in an isoproterenol-induced cardiac hypertrophy model in rats (Kitagawa et al., 2007) or in a rat model of I/R (Aune et al., 2014), while a number of other studies reported cardioprotection of TSA against isoproterenol or TAC-induced cardiac hypertrophy in mice, I/R injury in mice, or MI injury in mice (Kook et al., 2003; Kong et al., 2006; Zhang et al., 2012a; Zhang et al., 2012b; Ooi et al., 2015; Zhang L. et al., 2018). In a different study, administration of TSA exacerbated aortic calcification in a model of vitamin D3-induced vascular calcification (Kwon et al., 2016). The differences in administration conditions or the disease models may underlie the discrepancies. Therefore, it is critical to dissect how each HDACi impacts cardiac function under specific conditions, which might be dependent on the dose, route, duration, or disease models. Other adverse side effects of HDACi in clinical trials include disruption of the gastrointestinal and hematologic systems (Subramanian et al., 2010). Novel delivery methods to enrich HDACi in the heart might help reduce some side effects on other organs. However, arrhythmias were observed in some trials (Shah et al., 2007). A more comprehensive characterization of HDACi toxicity for chronic treatment will be necessary to evaluate their potential as a therapy for heart diseases.

Genetic manipulations of HDACs can have inconsistent effects as HDACi. Overall, there is more evidence supporting the detrimental effects of complete HDAC depletion on the heart, which is opposite to the overall cardioprotective effects of HDACi. First, this discrepancy could partially be attributed to differential developmental effects. Genetic KO of HDAC in the heart using constitutional Cre recombinase might disrupt developmental processes, whereas the administration of HDACi in adult animals would not. Inducible HDACs KO in adult animals in future studies can efficiently address this issue due to confounding developmental defects. Second, differential gene dosages might also contribute to the opposing effects of genetic HDAC depletion *versus* HDAC inhibition by HDACi. While a complete loss-of-function of HDAC might be detrimental to the heart, partial inhibition of HDACs by HDACi can be cardioprotective. It is possible that the abundance or function of HDACs needs to be tightly regulated according to the physiological context. For example, both cardiomyocyte-specific KO of HDAC3 and cardiomyocyte-specific transgenic OE of HDAC3 can lead to cardiac hypertrophy (Montgomery et al., 2008; Trivedi et al., 2008; Sun et al., 2011). Future studies comparing cardiac phenotypes by different abundance of HDACs in the heart can address such dose-dependent effects of HDACs. Third, previous studies have revealed the deacetylase-independent functions of HDACs (Sun et al., 2013). Thus, dissecting the deacetylase-independent *versus* deacetylase-dependent functions of HDAC will help provide additional insights into the different effects of genetic HDAC KO *versus* HDACi. Last, HDACi acts on the whole body, which makes it possible that the cardioprotection can be indirectly conferred through other organs in the body. In this case, future studies to further explore more tissue-specific roles of HDAC outside of the heart will help to understand how HDACs in other organs indirectly regulate cardiac function.

Omics analysis is a powerful tool for non-biased profiling of molecular changes caused by HDAC KO or HDACi treatment. However, the data does not distinguish direct *versus* indirect effects and does not suggest a causal role of gene expression changes in phenotypical changes. Further functional studies are warranted to dissect to what degree certain molecular changes are responsible for the observed cardiac phenotypic changes. Finally, it is also important to take into account how different HDACs can regulate or compensate one another in the heart. Future studies addressing the functional interaction of HDACs or simultaneous targeting of a combination of HDACs can be helpful in understanding their roles in heart diseases.
